# Dispersal corridors for plant species in the Poyang Lake Basin of southeast China identified by integration of phylogeographic and geospatial data

**DOI:** 10.1002/ece3.2999

**Published:** 2017-06-06

**Authors:** Dengmei Fan, Zhixia Sun, Bo Li, Yixuan Kou, Richard G. J. Hodel, Zhinong Jin, Zhiyong Zhang

**Affiliations:** ^1^ Laboratory of Subtropical Biodiversity Jiangxi Agricultural University Nanchang Jiangxi China; ^2^ Department of Biology University of Florida Gainesville FL USA; ^3^ Nanchang Institute of Technology Nanchang Jiangxi China

**Keywords:** chloroplast haplotype, dispersal corridor, Jiangxi Province, least cost path, Poyang Lake Basin, species distribution modeling

## Abstract

Measuring the dispersal of wildlife through landscapes is notoriously difficult. Recently, the categorical least cost path algorithm that integrates population genetic data with species distribution models has been applied to reveal population connectivity. In this study, we use this method to identify the possible dispersal corridors of five plant species (*Castanopsis tibetana*,* Schima superba*,* Cyclocarya paliurus*,* Sargentodoxa cuneata*,* Eomecon chionantha*) in the Poyang Lake Basin (PLB, largely coinciding with Jiangxi Province), China, in the late Quaternary. The results showed that the strongest population connectivity for the five species occurred in the Wuyi Mountains and the Yu Mountains of the eastern PLB (East Corridor) during the late Quaternary. In the western PLB, populations of the five species were connected by the Luoxiao Mountains and the Jiuling Mountains (West Corridor) but with a lower degree of connectivity. There were some minor connections between the eastern and the western populations across the Gannan Hills. When the corridors of five species were overlaid, the East Corridor and the West Corridor were mostly shared by multiple species. These results indicate that plant species in the PLB could have responded to the Quaternary climate changes by moving along the East Corridor and the West Corridor. Given that dispersal corridors have seldom been considered in the governmental strategies of biodiversity conservation in the PLB, preserving and restoring natural vegetation along these corridors should be prioritized to mitigate the effects of anthropogenic climate change by facilitating migration of plant species and other biota.

## 1 Introduction

A large body of scientific evidence suggests that climate on earth is changing rapidly (IPCC 2014). Climate change poses a challenge to conventional approaches of biodiversity conservation that rely on fixed protected areas (Williams et al., 2005), because the ranges of plants and animals are shifting in response to recent changes in climate (Parmesan & Yohe, 2003). Wildlife dispersal corridors may mitigate the effects of rapid climate change by allowing migration of biota (Epps, Wehausen, Bleich, Torress, & Brashares, 2007; Hobbs, 1992). However, evaluating and mapping wildlife dispersal corridors is challenging because ongoing dispersal of organisms through landscape is notoriously difficult to measure (Lowe & Allendorf, 2010).

However, climate change is not new for life on earth. In particular, there were substantial climate changes during the glacial–interglacial cycles of the Quaternary (Hewitt, 2000). Many species experienced repeated expansions and contractions in their ranges during the Quaternary (Davis & Shaw, 2001), leaving footprints that can be traced by fossil records or population genetic markers (Comes & Kadereit, 1998). Thus, locating historical dispersal corridors through tracing the footprints of past dispersal events may provide guidance for biodiversity conservation in the face of recent climate change (Brown & Yoder, 2015).

Phylogeography is the study of historical processes that may be responsible for the contemporary geographic distributions of individuals (Avise, 2000). As it analyzes the spatial distribution of genealogical lineages, phylogeography is a powerful tool for inferring historical recolonization routes (i.e., dispersal corridors) (Taberlet, Fumagalli, Wust‐Saucy, & Cosson, 1998). This approach is especially fruitful in flowering plants, because chloroplast DNA (cpDNA) does not recombine and is generally transmitted through seeds in angiosperms (Schaal, Hayworth, Olsen, Rauscher, & Smith, 1998); therefore, colonization patterns inferred from cpDNA are typically not blurred by pollen flow (Dumolin‐Lapègue, Demesure, Fineschi, Corre, & Petit, 1997). However, the hypothesized dispersal corridors in phylogeographic studies rely mostly on anecdotal biogeographic inferences (i.e., visual inspection of haplotype distribution) that often ignore the influence of geography and climate on organismal distribution (Crawford, Bermingham, & Carolina, 2007). In addition, the resulting dispersal corridors that are represented by lines and arrows are essentially subjective and thus difficult for other authors to replicate (e.g., Dumolin‐Lapègue et al., 1997; Taberlet et al., 1998; Tian et al., 2015). Recently, Chan, Brown, and Yoder (2011) developed a statistically rigorous method (the categorical least cost path, CLCP) by merging genetic and geospatial data to calculate historical dispersal corridors. The assumptions behind CLCPs are as follows: (1) a high probability of occurrence in species distribution models (SDMs) has a low cost to dispersal through the landscape matrix; (2) populations with shared (and/or sister) haplotypes likely experienced dispersal between sample localities. Thus, if we obtained all the shared (and/or sister) haplotypes between populations and past species distribution, it would be feasible to *calculate* rather than *infer* historical dispersal corridors of plant species across the landscape during the Quaternary using ArcGIS tools (Chan et al., 2011; Yu et al., 2015).

**Figure 1:**
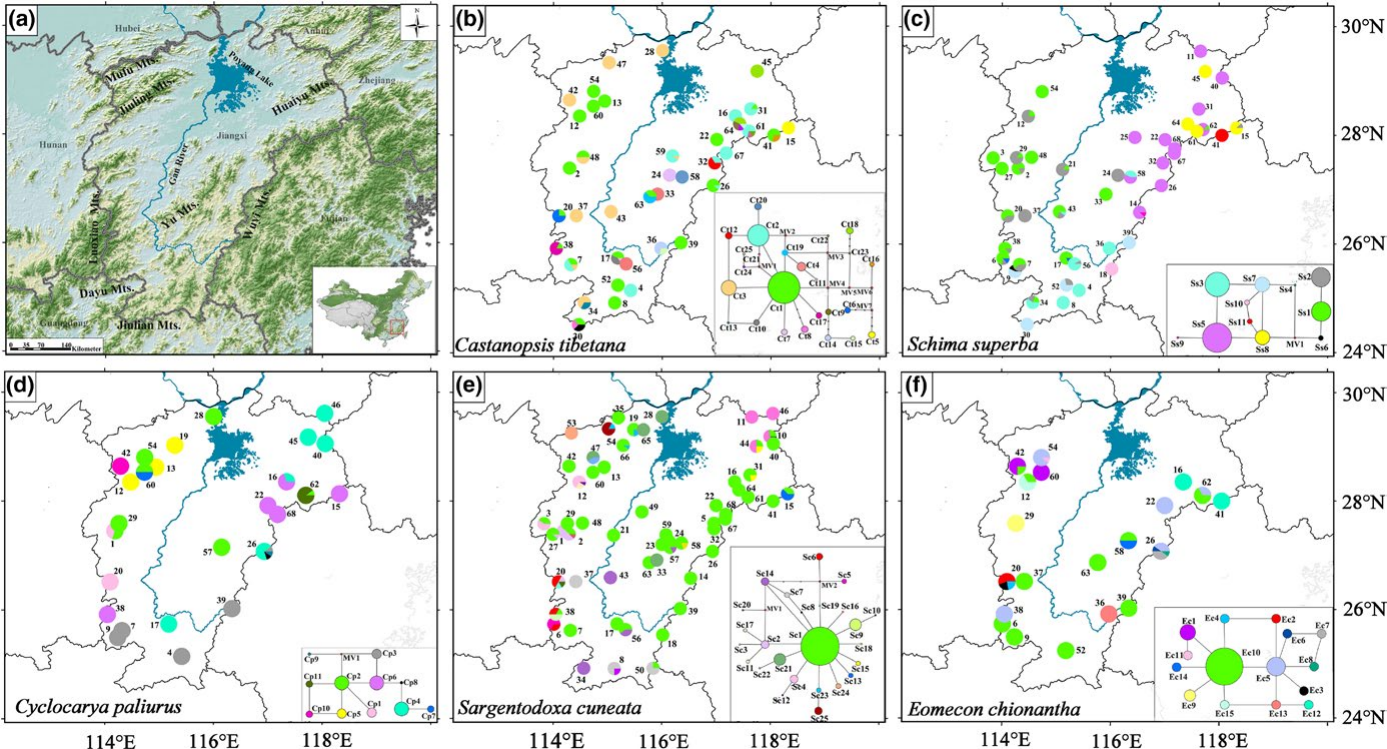
(a) Geographic characteristics of the Poyang Lake Basin. (b–f) Geographic distribution and median‐joining network of cpDNA haplotypes in *Castanopsis tibetana* (Ct1–Ct25), *Schima superba* (Ss1–Ss11), *Cyclocarya paliurus* (Cp1–Cp11), *Sargentodoxa cuneata* (Sc1–Sc25), and *Eomecon chionantha* (Ec1–Ec15), respectively. For each network, the size of circles corresponds to the frequency of each haplotype. Red dots indicate unsampled or extinct haplotypes. Each black line represents one mutational step that interconnects two haplotypes

Situated in the middle and lower reaches of the Yangtze River and largely coinciding with the Jiangxi Province, the Poyang Lake Basin (PLB) is a natural geographical unit in subtropical China (Figure 1a). Mountains surround the PLB on three sides, with the Mufu Mountains, the Jiuling Mountains, and the Luoxiao Mountains lying to the west, the Huaiyu Mountains and the Wuyi Mountains (including the Yu Mountains) to the east, and the Jiulian Mountains and the Dayu Mountains to the south. The Gan River bisects the PLB, flowing through the basin from south to north. In the north, it enters the Poyang Lake, the largest freshwater lake in China. The lake in turn empties into the Yangtze River, which forms part of the northern border of the PLB. The southern half of the PLB is hilly with ranges and valleys interspersed throughout its territory (referred to as Gannan Hills), while the northern half is flatter and lower in altitude (Figure 1a; Liu, 2008).

The PLB has a subtropical humid monsoon climate (mean annual temperature of 16–20°C and annual rainfall of 1,400–1,900 mm), high forest cover (63.1%, ranking second in China) and is rich in biodiversity (official website of Jiangxi Provincial Department of Forestry, http://www.jxly.gov.cn/). Because the government of Jiangxi province is dedicated to wildlife and environmental protection, 188 nature reserves (including 15 national nature reverses) have been established since the 1980s (http://www.jxly.gov.cn/). However, functional connectivity among nature reserves has never been incorporated in conservation practices, possibly due to conventional views on protected areas and limited knowledge about dispersal corridors. Recently, Tian et al. (2015) identified two recolonization routes for *Sargentodoxa cuneata* (Lardizabalaceae) along the Wuyi Mountains and the Luoxiao Mountains, respectively. However, the recolonization routes (dispersal corridors) identified in their study was difficult to replicate because their inference was based on a subjective inspection of haplotype distribution. In addition, conservation strategies that simultaneously meet the needs of multiple species may be more effective for broad‐spectrum biodiversity conservation (Cushman & Landguth, 2012), evaluating dispersal corridors for multiple species should be of higher priority. However, such studies in the PLB remain scant.

In this study, we first characterized the phylogeographic structure of five plant species in the PLB (including *S. cuneata*, with increased population sampling within the basin). Then, dispersal corridors for each species during the late Quaternary were constructed using the CLCP method of Chan et al. (2011). Finally, we overlaid the migration corridors of five species to identify shared corridors. The results of this study would provide an illustration of how plant species have responded to climate changes during the Quaternary in the PLB and optimize the conservation strategies under ongoing climate change.

## 2 Materials and methods

### 2.1 Plant species and population sampling

Five common plant species in the PLB, *Castanopsis tibetana* (Fagaceae), *Schima superba* (Theaceae), *Cyclocarya paliurus* (Juglanaceae), *S. cuneata* (Lardizabalaceae), *Eomecon chionantha* (Papaveraceae), were selected for two reasons. First, except for *E. chionantha*, all species have been phylogeographically investigated across their distribution ranges (Fan et al., 2016; Kou et al., 2016; Tian et al., 2015); thus, it is easier to get a dense sampling in the PLB. Second, these species represent a broad spectrum of growth habits, dispersal modes of seeds (or fruits), and other ecological characteristics. *Castanopsis tibetana* and *S. superba* are representatives of evergreen forest trees, *C. paliurus* is a deciduous broad‐leaved tree, *S. cuneata* is a woody liana*,* and *E. chionantha* is a perennial herb. The dispersal modes of the five species vary from wind‐dispersed (*S. superba* and *C. paliurus*), animal‐cached (*C. tibetana*), animal‐ingested (*S. cuneata*), to explosive dehiscence (*E. chionantha*) (personal observation by Z.Y. Zhang). Species that belong to monotypic (*C. paliurus*,* S. cuneata*,* E. chionantha*) or oligotypic genera (*S. superba*) were favored to avoid the influence of interspecific hybridization as much as possible.

We conducted a comprehensive field survey in the PLB and investigated every county in the basin. In total, 39 (18), 44 (18), 26 (21), 58 (17), and 20 populations were sampled for *C. tibetana*,* S. superba*,* C. paliurus*,* S. cuneata*, and *E. chionantha*, respectively (Table S1 and Figure 1, the numbers in the parentheses are the sampled populations in the PLB in our previous phylogeographic studies, Tian et al., 2015; Kou et al., 2016; Fan et al., 2016). Because humans have lived in the PLB for thousands of years and native vegetation in many low elevation areas (especially in areas around Poyang Lake) has been severely depleted, the population sampling is somewhat uneven and biased toward the mountains and higher elevation areas (Figure 1). In each population, we sampled fresh leaves from at least six individuals when possible. All samples were desiccated in silica gel and stored at −20°C until being processed. Genomic DNA was extracted using a modified CTAB procedure (Doyle & Doyle, 1987).

### 2.2 Determination of shared and sister haplotypes

The chloroplast DNA markers (two intergenic spacers for each species) and experimental procedure of *C. tibetana*,* S. superba*,* C. paliurus,* and *S. cuneata* were the same as in the previous studies (Fan et al., 2016; Kou et al., 2016; Tian et al., 2015). For *E. chionantha*, two intergenic spacers, *trn*C‐*rpo*B (F: CACCCRGATTYGAACTGGGG; R: CKACAAAAYCCYTCRAATTG; Shaw, Lickey, Beck, & Small, 2007) and atpB‐rbcL (F: ACACCAGCTTTGAATCCAAC; R: AGAACCAGAAGTAGTAGGAT; Manos & Stone, 2001) were selected. Amplification reactions were carried out in a volume of 20 μl containing 10 μl 2× *Taq* PCR MasterMix (Tiangen, Shanghai, China), 1 μl each forward and reverse primer (0.2 umol/l), 1 μl template DNA (*ca*. 50–100 ng) and 7 μl ddH_2_O. Amplification was carried out in a Bioer XP cycler (Bioer, Hangzhou, China) programmed for an initial 3‐min denaturation at 94°C, followed by 33 cycles of 30‐s denaturation at 94°C, 30‐s annealing at 50°C (*trn*C‐*rpo*B) or 53°C (*atp*B‐*rbc*L), 1 min extension at 72°C, and a final 5‐min extension at 72°C. Sequencing reactions were performed with the corresponding forward and reverse primers commercially by Sangon Biotech Co., Ltd. (Shanghai, China).

According to the CLCP method of Chan et al. (2011), putative dispersal corridors are determined by shared or sister haplotypes. Therefore, the chloroplast sequences of the same species were concatenated to define cpDNA haplotypes. The haplotypes detected in our previous studies were pooled with the new data set. A haplotype network was inferred for each species under the criterion of statistical parsimony using Network 4.6 (Bandelt, Forster, & Rohl, 1999), with indels being coded as substitutions in *E. chionantha* or following the treatments in previous phylogeographic studies.

### 2.3 Species distribution modeling

We employed the maximum entropy approach (MAXENT, Phillips, Anderson, & Schapire, 2006) to predict the distribution of the five species in the PLB at the present and at the time of the LGM (0.021–0.018 Ma). We compiled nineteen environmental variables for the time of the LGM (MIROC 3.2 scenario; with a resolution of 2.5 arc min) and for the present (with a resolution of 30 arc seconds) from the WORLDCLIM database (http://www.worldclim.org, Hijmans, Cameron, Parra, Jones, & Jarvis, 2005) for each environmental layer.

Species distribution models were constructed using 50/53/42/63/40 presence records of *C. tibetana*,* S. superba*,* C. paliurus*,* S. cuneata*, and *E. chionantha* in the PLB, respectively, including all sites recorded during our field work and publicly available specimen records with GPS data (Chinese Virtual Herbarium). We used the default parameters of MAXENT and included 80% of species records for training and 20% for testing the model. The ability of the model to predict the presence or absence of a species was measured by the area under the “receiver operating characteristic (ROC) curve” (AUC; Fielding & Bell, 1997; Elith et al., 2006). A score between 0.7 and 1.0 indicates that the model performs better than random and was considered acceptable discrimination (Fielding & Bell, 1997). To measure the similarity of SDMs between the LGM and the present for each species, we calculated Schoener's *D* using ENMTOOLS version 1.3 (Warren, Glor, & Turelli, 2008).

### 2.4 Visualizing dispersal corridors

For each species, the dispersal corridors at the present and at the LGM were mapped by applying the CLCP method using SDMtoolbox (Brown, 2014) in ArcGIS 9.3 (Environmental Systems Research Institute, Inc., Redlands, CA). Given a chloroplast haplotype network, we generated a population connectivity map by summing the least cost paths (LCPs) among all shared and sister haplotypes from different localities in ArcGIS using the dispersal cost as the friction layer. Then, we obtained a dispersal cost layer (resistance layer) by inverting the SDMs (i.e., calculating 1‐SDM), and subsequently we created a cost distance raster for each sample locality using the resistance layer. Based on the cost distance raster, corridor layers were established between two localities based on shared and sister haplotypes. To avoid oversimplifying landscape processes, we classified the LCPs into four categories: lowest value–1% LCPs, 1%–2% LCPs, 2%–5% LCPs, and 5% LCPs–maximum value. Next, these four categories were reclassified as new values: 5, 2, 1, and 0, respectively (Chan et al., 2011). Finally, we summed up and standardized all of the pairwise reclassified corridor layers and identified dispersal maps of the five species in an explicit landscape. The final dispersal map for each species was divided into classes by the classification method of standard deviation, which had a middle class centered on the mean with a range of 1 standard deviation (0.5 standard deviation to either side of the mean) (Brewer & Pickle, 2002). And the values in the dispersal network greater than 0.5 standard deviations from the mean were considered areas with the highest connectivity, which is similar to the method of Vandergast, Bohonak, Hathaway, Boys, and Fisher (2008). Shared corridors by the five species were determined by intersecting the dispersal network greater than 0.5 standard deviations of each species.

## 3 Results

### 3.1 Haplotype distribution of five plant species in the PLB

Twenty‐five, 11, 11, 25, and 15 chloroplast haplotypes were identified in *C. tibetana*,* S. superba*,* C. paliurus*,* S. cuneata*, and *E. chionantha* in the PLB, respectively (Table S1 and Figure 1b–f). Among them, 12, 5, 3, and 11 new haplotypes were recovered for *C. tibetana*,* S. superba*,* C. paliurus*,* S. cuneata*, respectively, because much more populations were sampled in the PLB than in previous studies. All newly produced haplotype sequences were deposited in GenBank (accession numbers: KX868108–KX868281). The haplotype distribution patterns of the five species were quite different. Geographical structure of lineages in *C. tibetana* and *S. cuneata* was not obvious because each species contained a widespread haplotype (Ct1 and Sc1, Figure 1b,e). In contrast, the haplotype distribution in *S. superba*,* C. paliurus* and *E. chionantha* was more structured. For example, all five of the most frequent haplotypes of *C. paliurus* (Cp1–Cp5, Figure 1d) were regionally restricted and an east–west division appeared in *S. superba* (Figure 1c). The haplotype relationships for each species were complex without any obviously differentiated clades (Figure 1b–f).

### 3.2 Potential distribution of five species in the PLB since the LGM

**Figure 2:**
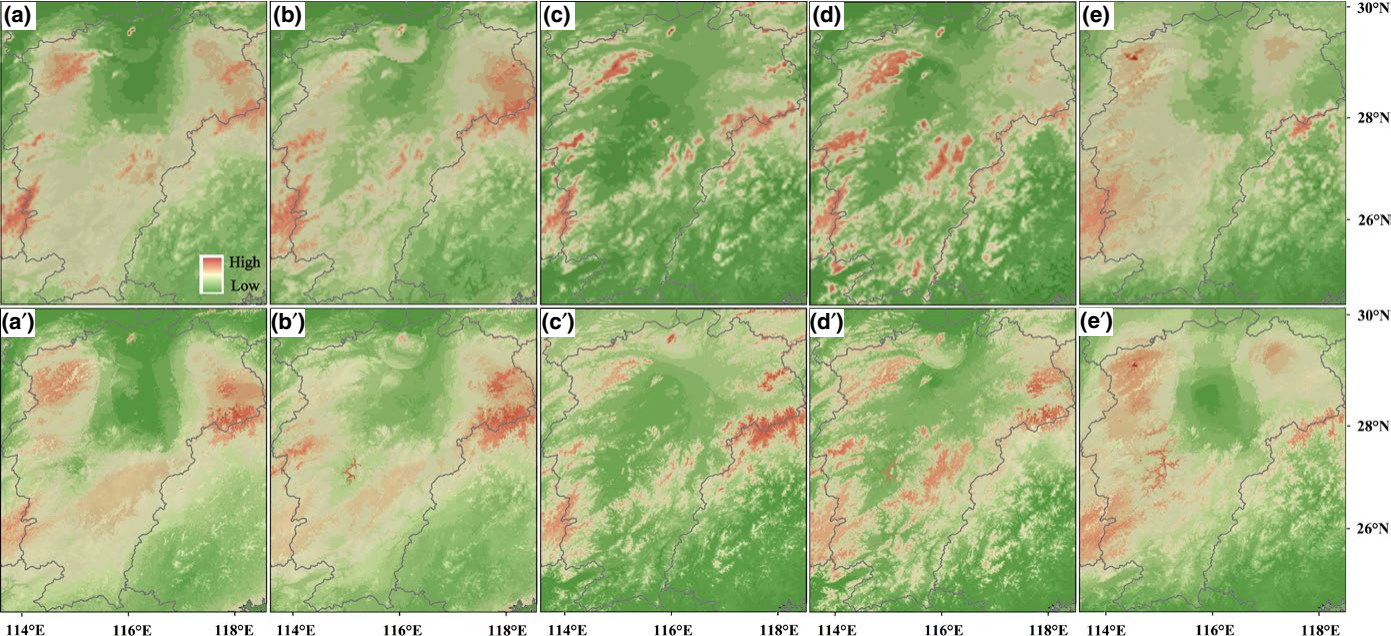
(a–e) Predicted distributions based on species distribution modeling at the LGM and (a′–e′) at the present for *Castanopsis tibetana*,* Schima superba*,* Cyclocarya paliurus*,* Sargentodoxa cuneata,* and *Eomecon chionantha*, respectively. Warm colors represent areas of higher habitat suitability

At the present and the LGM, the areas under the “receiver operating characteristic (ROC) curve” (AUC) values were 0.811/0.806, 0.788/0.807, 0.823/0.858, 0.814/0.842, and 0.797/0.806 for *C. tibetana*,* S. superba*,* C. paliurus*,* S. cuneata*, and *E. chionantha*, respectively, indicating good predictive model performance. At the LGM, the potential ranges of the five species were very similar to the ranges at the present (Schoener's *D* values were 0.911, 0.930, 0.915, 0.885, and 0.914 for *C. tibetana*,* S. superba*,* C. paliurus*,* S. cuneata*, and *E. chionantha*, respectively, and also see Figure 2), suggesting these species survived in the PLB since the LGM as indicated in our previous study (Fan et al., 2016). Intuitively, these results are prone to the interpretation of overall range stability, with most populations remaining in place. Range stability is often inferred by SDMs for plant species in subtropical China; however, most species have experienced complex range shifts during the late Quaternary (e.g., Fan et al., 2016; Gong et al., 2016) because the climate in subtropical China since the LGM has undergone profound changes (Zheng, Yuan, & Petit‐Maire, 1998).

### 3.3 Dispersal corridors at the LGM and at the present time

**Figure 3:**
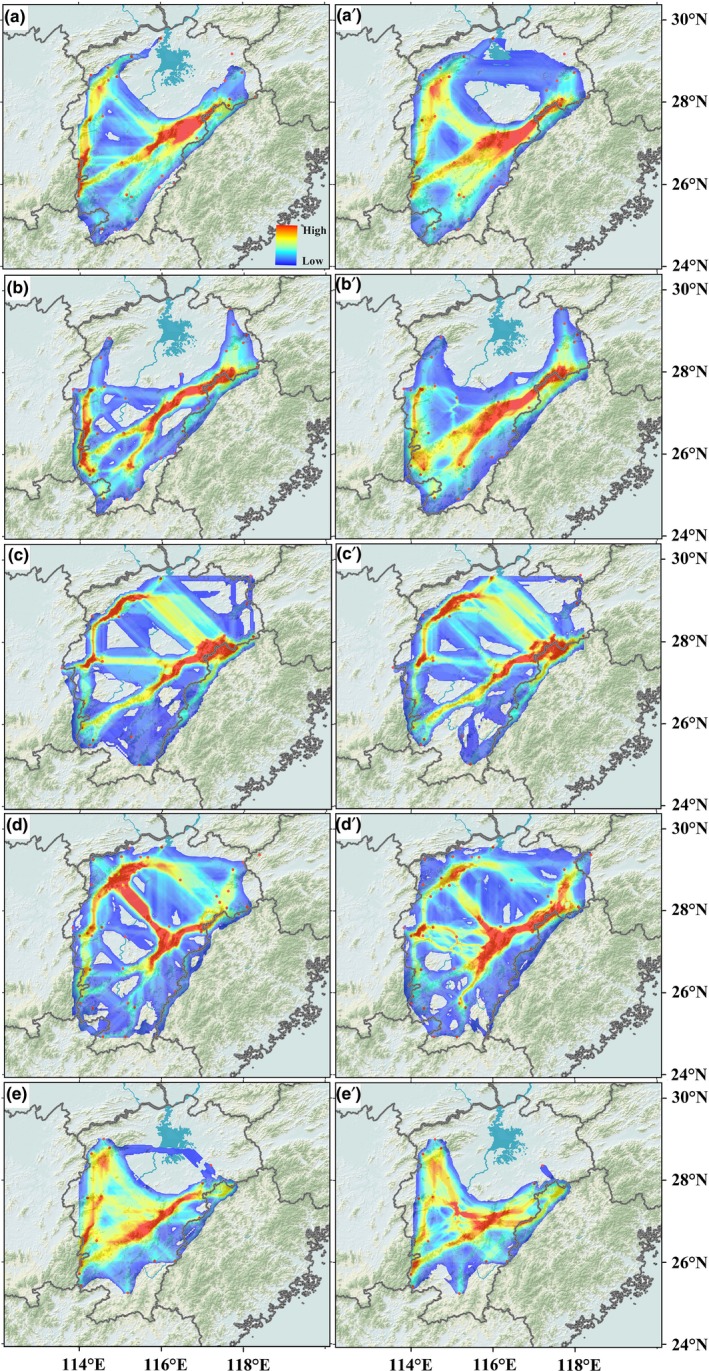
(a–e) Potential dispersal corridors at the LGM and (a′–e′) at the present of *Castanopsis tibetana*,* Schima superba*,* Cyclocarya paliurus*,* Sargentodoxa cuneata,* and *Eomecon chionantha*, respectively. Population connectivity ranges from its highest values in red to its lowest values in blue

**Figure 4:**
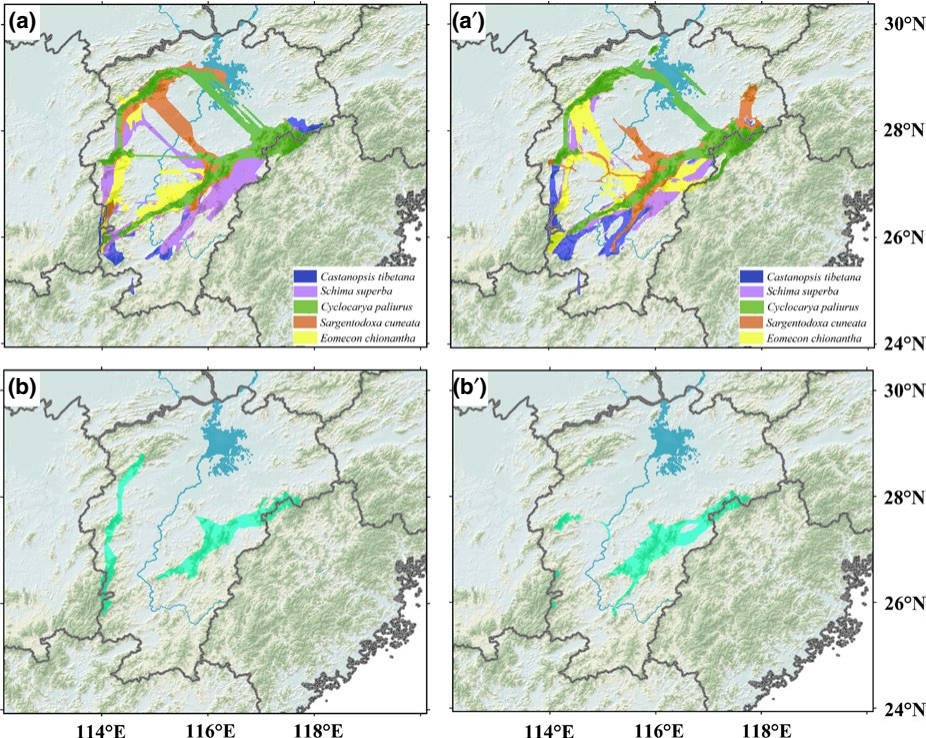
(a) Dispersal corridors of five plant species at the LGM and (a′) at the present; (b) shared dispersal corridors by five plant species at the LGM and (b′) at the present

Both at the LGM and at the present, the strongest population connectivity for the five species occurred in the Wuyi Mountains and the Yu Mountains of the eastern PLB, indicating a southwest–northeast dispersal corridor (Figures 3 and 4a,a′). In the western PLB, populations of the five species were also connected by the Luoxiao Mountains but with a lower degree of connectivity (Figures 3 and 4a,a′). The Jiuling Mountain in the northwest PLB was a putative dispersal corridor for *C. paliurus* and *E. chionantha,* which could be an extension of the corridor of Luoxiao Mountains (Figures 3 and 4a,a′). There were some connections between the east and west populations, but these connections mostly occurred in the middle and south PLB and were absent or very weak in the north (Figure 4a,a′). When the corridors of five species were intersected, the east corridor of Wuyi Mountains and Yu Mountains was mostly shared by multiple species throughout the late Quaternary. In contrast, the west corridor of Luoxiao Mountains and Jiuling Mountains occurred for multiple species at the LGM but almost disappeared at the present (Figure 4b,b′).

## 4 Discussion

### 4.1 Two major dispersal corridors for plant species in the PLB

Inferring the locations of glacial refugia and recolonization routes (dispersal corridors) has been one of the major objectives of phylogeography (Comes & Kadereit, 1998). However, traditional phylogeographic approaches rarely consider the influence of geography and climate on organismal distribution and do not permit visualizing recolonization routes objectively. The CLCP method (Chan et al., 2011) identified two major dispersal corridors for multiple plant species in the PLB since the LGM: one situated in the eastern PLB (East Corridor), extending from the Yu Mountains or the lower Wuyi Mountains to the upper Wuyi Mountains, another (West Corridor) from the Luoxiao Mountains to the Jiuling Mountains. In addition, several minor dispersal corridors linking the east and west populations of the PLB were also detected (Figures 3 and 4).

Extending in a northeast–southwest direction, the Wuyi Mountains and adjacent Mountains have long been viewed as a dispersal corridor for plant species in China (Wang, 1992; Fig. 21). However, Wang (1992) did not provide a detailed description of the corridor. Recently, other researchers have referenced this corridor in several phylogeographic studies. For example, Chen, Compton, Liu, and Chen (2012) found that a clade of pollinators of *Ficus pumila* expanded its range northward along the Wuyi Mountains during the Holocene. Based on a phylogeographic analysis of range‐wide samples, Tian et al. (2015) found that the Wuyi Mountains were one major dispersal corridor for *S. cuneata*. However, Chen et al. (2012) did not report the exact location of the corridor and Tian et al. (2015) only diagrammed the corridor with arrows. With more intensive sampling within the PLB and the geospatially explicit CLCP method, a corridor extending from the Yu Mountains to the upper Wuyi Mountains was described for *S. cuneata* in our study (Figure 3d,d′). Moreover, our study found that the Wuyi Mountains and adjacent mountains act as a dispersal corridor not only for *S. cuneata,* but also for other focal species (Figures 3 and 4), highlighting the importance of this corridor for plant species in southeast China during the Quaternary climate oscillations.

The Luoxiao Mountains largely parallel the Wuyi Mountains, but Wang (1992) did not consider the Luoxiao Mountains as a potential corridor in the north–south direction. Additionally, few phylogeographic studies identified this dispersal corridor for plant species in southeast China except for Tian et al. (2015). The failure to detect the Luoxiao Mountains as a corridor may be explained by either inadequate population sampling within the Mountains or that the Luoxiao Mountains are not a corridor for plant species. The former explanation is preferred because this study found multiple species that utilize these mountains as a corridor, although the degree of connectivity is lower than in the East Corridor (Figures 3 and 4a,a′). In fact, several phylogeographic studies on some plant species, such as *Pteroceltis tatarinowii* (Ulmaceae) (Li, Shao, Lu, Zhang, & Qiu, 2012) and *Loropetalum chinense* (Hamamelidaceae) (Gong et al., 2016), found that extensive inter/postglacial expansion had happened prevalently in east China (including the Luoxiao mountains). However, sparse sampling within the Luoxiao Mountains prevented the corridor from being discovered until recently. In this study, we found that the Luoxiao Mountains (along with the Jiuling Mountains) acted as a corridor by all the five species at the LGM. At the present, the corridor occurs in individual species (Figure 3a′–e′). The absence of the shared West Corridor may be owing to little intersection within the Luoxiao Mountains and the Jiuling Mountains among the five species.

Although two major corridors in the PLB were identified, the exact locations of corridors are quite different among the plant species. For example, the lower Wuyi Moutains act as a corridor for *C. tibetana* (Figure 3a,a′) and *S. superba* (Figure 3b,b′), but not for the other species. The east–west population connectivity is stronger in *C. paliurus* (Figure 3c,c′) and *S. cuneata* (Figure 3d,d′) than in the other three species. These differences are consistent with the results of similar studies in other areas (e.g., Soltis, Morris, McLachlan, Manos, & Soltis, 2006; Taberlet et al., 1998). For example, Taberlet et al. (1998) found only a small degree of congruence among the likely postglacial colonization routes of ten species in Europe. The focal species of this study belong to different families and vary in dispersal mode and environmental tolerance. Therefore, incongruence in dispersal corridors among species in the PLB may reflect the individualistic responses of species to climate change that depend on specific adaptations and dispersal modes of individual species (Stewart, Lister, Barnes, & Dalén, 2010).

### 4.2 Implications for plant evolutionary history and biodiversity conservation in the PLB

Characterized by a mild Asian monsoon climate, subtropical China (including the PLB) was not glaciated during the Quaternary (Zheng et al., 1998). Multiple glacial refugia and limited inter/postglacial expansions have been suggested as a paradigm for plant species in this region (reviewed by Liu, Sun, Ge, Gao, & Qiu, 2012). However, a growing number of studies indicated that extensive inter/postglacial expansions may be the rule for plant species, and not the exception, as the region underwent profound climate changes throughout the Quaternary. For example, the climate in subtropical China was cooler by c. 4–6°C in mean annual temperature and drier by c. 400–600 mm/year in annual precipitation according to pollen records from the LGM (Zheng et al., 1998). In this study, widespread haplotypes were found in different species, implying that range expansions could have happened since the LGM. Thus, determining the locations of dispersal corridors can provide important insights into the range shift histories of plant species and also guide the conservation of biodiversity in the PLB in the face of global climate change.

Improving species’ ability to cope with climate change is a major strategy for China's national biodiversity conservation efforts (Ministry of Environmental Protection of the People's Republic China, 2011). Construction of dispersal corridors is an effective way to mitigate the effects of rapid climate change by allowing biota to migrate (Epps et al., 2007; Hobbs, 1992). Since the first nature reserve was built in the PLB in 1981, 188 nature reserves, accounting for 7.1% of land area of Jiangxi Province, have been established by governments at different administrative levels (http://www.jxly.gov.cn/). However, thus far, dispersal corridors have not been designed, or even conceived, for the functional connectivity of different nature reserves in the PLB. In this study, we identify two major dispersal corridors for plant species in the PLB throughout the late Quaternary. As this study investigated only five plant species and the samples biased toward mountainous areas, it is highly likely that more dispersal corridors (including lowland corridors) would be discovered if more organisms were studied with denser sampling. In spite of this caveat, we still believe that most plant species in the basin would expand toward the north along the two major corridors in the face of the ongoing global warming, because the two dispersal corridors are mostly congruent among multiple species with a broad range of ecological characteristics. In addition, a recent study on the geographical distribution of endangered plant species in Jiangxi Province suggested that connectivity among isolated populations of *Ulmus elongata* (Ulmaceae)*, Torreya grandis* (Taxaceae)*, Disanthus cercidifolius* var. *longipes* (Hamamelidaceae) is possibly maintained by the Wuyi Mountains, the Luoxiao Mountains, and their adjacent mountains (Yu, 2016), supporting the existence of two major corridors. Thus, we strongly recommend that preserving and restoring natural vegetation along those corridors should be incorporated into provincial conservation policy and practices, in addition to the establishment of fixed protected areas. This strategy will facilitate the northward migration of plants as well as other biotas, such as animals, and mitigate the adverse effect of global warming in the densely populated PLB with fragmented natural vegetation. Furthermore, the Gannan Hills are important for connectivity between east and west populations and thus should also be prioritized when habitat corridors are established. In contrast, Poyang Lake and its surrounding lowlands represent a barrier to dispersal in most plants; linking the east and west populations of the lake could be unnecessary.

## Conflict of interest

None declared.
