# Supplementary material for: Dispersal corridors for plant species in the Poyang Lake Basin of southeast China identified by integration of phylogeographic and geospatial data
Source: Ecol Evol. 2017 Jun 6;7(14):5140–8. doi: 10.1002/ece3.2999 (PMC5528243; doi:10.1002/ece3.2999)
Supplement: Supplementary file 1 [file ECE3-7-5140-s001.doc]

Table S1. Collection localities, coordinates, sample size and haplotypes recovered for the 39 *Castanopsis tibetana*, 44 *Schima superba*, 26 *Cyclocarya paliurus*, 58 *Sargentodoxa cuneata*, 20 *Eomecon chionantha* populations investigated in Jiangxi.

| Population  ID | sampling  sites | Latitude (°N)/  Longitude (°E) | | *Castanopsis tibetana* | | |  | | *Schima superba* | |  | *Cyclocarya paliurus* | |  | *Sargentodoxa cuneata* | |  | *Eomecon chionantha* | |
| --- | --- | --- | --- | --- | --- | --- | --- | --- | --- | --- | --- | --- | --- | --- | --- | --- | --- | --- | --- |
|  |  |  |  | Haplotype | sample |  | | Haplotype | | sample |  | Haplotype | sample |  | Haplotype | sample |  | Haplotype | sample |
| 1 | Anfu1 | 27.45 /114.19 | | — | — |  | | — | | — |  | Cp1Cp | 10 |  | Sc1Sc2Sc3 | 6 |  | — | — |
| 2 | Anfu2 | 27.39/114.29 | | Ct1 | 8 |  | | Ss1 Ss2 | | 6 |  | — | — |  | Sc1Sc2Sc16 | 8 |  | — | — |
| 3 | Anyuan1 | 27.58/ 113.83 | | — | — |  | | Ss1 | | 8 |  | — | — |  | Sc1 Sc4 | 10 |  | — | — |
| 4 | Anyuan2 | 25.15/ 115.42 | | Ct2 | 7 |  | | Ss3 | | 11 |  | Cp3 | 1 |  | — | — |  | — | — |
| 5 | Chongren | 27.59 /116.96 | | — | — |  | | — | | — |  | — | — |  | Sc1 | 11 |  | — | — |
| 6 | Chongyi1 | 25.74/ 114.01 | | — | — |  | | Ss1 Ss2 Ss4 | | 7 |  | — | — |  | Sc5Sc6 | 6 |  | Ec10 | 3 |
| 7 | Chongyi2 | 25.62/ 114.32 | | Ct1 Ct2Ct3 | 6 |  | | Ss1 Ss2 | | 10 |  | Cp3 | 10 |  | Sc1 | 2 |  | — | — |
| 8 | Dingnan | 24.92/ 115.12 | | Ct1 | 5 |  | | Ss3 | | 10 |  | — | — |  | Sc7Sc8 | 4 |  | — | — |
| 9 | Dayu | 25.50/ 114.24 | | — | — |  | | Ss6 Ss7 | | 7 |  | Cp3 | 1 |  | — | — |  | Ec10 | 3 |
| 10 | Dexing | 29.19/ 118.00 | | — | — |  | | — | | — |  | — | — |  | Sc1Sc9Sc10 | 11 |  | — | — |
| 11 | Fuliang | 28.75/ 116.46 | | — | — |  | | Ss5 | | 8 |  | — | — |  | Sc9 | 5 |  | — | — |
| 12 | Fengyi | 28.35/ 114.48 | | Ct1 | 4 |  | | Ss1Ss2 | | 10 |  | Cp5 | 7 |  | Sc4Sc11Sc12 | 12 |  | Ec10Ec15 | 3 |
| 13 | Fengxin | 28.62/ 114.94 | | Ct1 | 6 |  | | — | | — |  | Cp5 | 7 |  | Sc1 | 8 |  | — | — |
| 14 | Guangchang | 26.58/ 116.53 | | — | — |  | | Ss5 Ss9 | | 8 |  | — | — |  | Sc1 | 6 |  | — | — |
| 15 | Guangfeng | 28.11 /118.09 | | Ct5 | 8 |  | | Ss2 Ss8 | | 8 |  | Cp6 | 10 |  | Sc1Sc13 | 8 |  | — | — |
| 16 | Guixi | 28.36 /117.35 | | Ct2 | 8 |  | | — | | — |  | Cp4Cp6 | 10 |  | Sc1 | 9 |  | Ec12 | 8 |
| 17 | Ganxian | 25.74/ 115.18 | | Ct1 Ct10 Ct11 | 7 |  | | Ss1 Ss4 | | 6 |  | Cp4 | 4 |  | Sc1 | 6 |  | — | — |
| 18 | Huichang | 25.54 /116.02 | | — | — |  | | Ss10 | | 8 |  | — | — |  | Sc1 | 2 |  | Ec2 Ec3Ec4 | 9 |
| 19 | Jing'an | 29.03 /115.29 | | — | — |  | | — | | — |  | Cp5 | 9 |  | Sc1Sc23 | 10 |  | — | — |
| 20 | Jinggangshan | 26.52/ 114.10 | | Ct1 Ct6 | 8 |  | | Ss1 Ss2 | | 10 |  | Cp1 | 10 |  | Sc2 Sc6 Sc19 Sc20 | 6 |  | — | — |
| 21 | Jishui | 27.37 /115.11 | | — | — |  | | Ss1 Ss2 | | 8 |  | — | — |  | Sc1 | 7 |  | — | — |
| 22 | Jinxi | 27.92 /117.88 | | Ct1 | 8 |  | | Ss5 | | 8 |  | Cp6 | 10 |  | Sc1 | 5 |  | Ec5 | 6 |
| 23 | Le'an1 | 27.20/ 116.00 | | — | — |  | | — | | — |  | — | — |  | Sc1 | 16 |  | — | — |
| 24 | Le'an2 | 27.24/ 116.01 | | Ct7 | 5 |  | | Ss2 | | 10 |  | — | — |  | Sc1 | 5 |  | — | — |
| 25 | Linchuan | 27.96 /116.45 | | — | — |  | | Ss5 | | 8 |  | — | — |  | — | — |  | — | — |
| 26 | Lichuan | 27.08 /116.94 | | Ct1Ct2 | 10 |  | | Ss5 | | 9 |  | Cp3Cp4Cp8Cp9 | 10 |  | Sc1 | 5 |  | Ec5Ec6Ec7Ec8 | 10 |
| 27 | Lianhua | 27.39 /114.00 | | — | — |  | | Ss1 | | 8 |  | — | — |  | Sc1Sc2 | 8 |  | — | — |
| 28 | Lushan | 29.55/ 116.01 | | Ct3 | 3 |  | | — | | — |  | Cp2 | 9 |  | Sc21 | 8 |  | — | — |
| 29 | Luxi | 27.59 /114.26 | | — | — |  | | Ss1 Ss2 | | 7 |  | Cp2 | 2 |  | Sc1 | 7 |  | Ec9 | 11 |
| 30 | Longnan | 24.52 /114.46 | | Ct1Ct8 Ct9 | 8 |  | | Ss7 | | 6 |  | — | — |  | — | — |  | — | — |
| 31 | Dexing2 | 28.48/ 117.63 | | Ct1 Ct2 | 7 |  | | Ss5 | | 7 |  | — | — |  | Sc1Sc15 | 9 |  | — | — |
| 32 | Nancheng | 27.49/ 116.96 | | Ct1 Ct12 | 6 |  | | Ss5 | | 8 |  | — | — |  | Sc1 | 11 |  | — | — |
| 33 | Ningdu | 27.19 /115.95 | | Ct4 | 9 |  | | Ss1 | | 9 |  | — | — |  | Sc21 | 5 |  | — | — |
| 34 | Quannan | 24.93/ 114.56 | | Ct3 Ct13 | 6 |  | | Ss1 Ss2 Ss3 | | 5 |  | — | — |  | Sc14 | 5 |  | — | — |
| 35 | Ruichang | 29.54 /115.20 | | — | — |  | | — | | — |  | — | — |  | Sc1 | 11 |  | — | — |
| 36 | Ruijin | 25.92/ 115.97 | | Ct14Ct15 | 7 |  | | Ss3 | | 11 |  | — | — |  | — | — |  | Ec13 | 4 |
| 37 | Suichuan | 26.57/ 114.25 | | Ct3 | 6 |  | | Ss2 | | 7 |  | — | — |  | Sc7 | 2 |  | Ec10 | 7 |
| 38 | Shangyou | 25.92/ 114.05 | | Ct1 Ct17 | 6 |  | | Ss1 Ss2 | | 8 |  | Cp6 | 10 |  | Sc1Sc6Sc17 | 6 |  | Ec5 | 4 |
| 39 | Shicheng | 26.02 /116.34 | | Ct1 | 8 |  | | Ss7 | | 8 |  | Cp3 | 5 |  | Sc1 | 11 |  | Ec10 | 8 |
| 40 | Sanqingshan | 29.06/ 118.06 | | — | — |  | | Ss5 | | 11 |  | Cp4 | 9 |  | Sc1 | 2 |  | — | — |
| 41 | Sahngrao | 28.07/ 118.11 | | Ct1Ct16 | 7 |  | | Ss11 | | 8 |  | — | — |  | Sc1 | 11 |  | Ec12 | 2 |
| 42 | Tonggu | 28.64 /114.29 | | Ct3 | 7 |  | | — | | — |  | Cp10 | 6 |  | Sc1 | 11 |  | Ec1Ec10 | 4 |
| 43 | Taihe | 26.59 /115.06 | | Ct3 | 2 |  | | Ss1 Ss2Ss3 | | 10 |  | — | — |  | Sc14 | 1 |  | — | — |
| 44 | Wuyuan1 | 29.01/ 117.74 | | — | — |  | | — | | — |  | — | — |  | Sc1Sc9Sc15 | 8 |  | — | — |
| 45 | Wuyuan2 | 29.18 /117.74 | | Ct18 | 6 |  | | Ss8 | | 8 |  | Cp4 | 10 |  | — | — |  | — | — |
| 46 | Wuyuan3 | 29.37 /118.39 | | — | — |  | | — | | — |  | Cp4 | 8 |  | Sc9 | 10 |  | — | — |
| 47 | Wuning | 29.33 /115.02 | | Ct3 | 6 |  | | — | | — |  | — | — |  | Sc23Sc25 | 12 |  | — | — |
| 48 | Wangzai | 27.60 /114.54 | | Ct1 Ct3 | 2 |  | | Ss1 | | 12 |  | — | — |  | Sc1 | 8 |  | — | — |
| 49 | Xingan | 27.80/ 115.64 | | — | — |  | | — | | — |  | — | — |  | Sc1 | 5 |  | — | — |
| 50 | Xunwu | 24.92 /115.84 | | — | — |  | | — | | — |  | — | — |  | Sc1Sc7 | 4 |  | — | — |
| 51 | Xinguo | 26.49 /115.78 | | — | — |  | | — | | — |  | — | — |  | — | — |  | — | — |
| 52 | Xinfeng | 25.71/ 114.00 | | Ct1 | 8 |  | | Ss2Ss7 | | 7 |  | — | — |  | — |  |  | Ec10 | 9 |
| 53 | Xiushui1 | 29.25/ 114.34 | | — | — |  | | — | | — |  | — | — |  | Sc24 | 11 |  | — | — |
| 54 | Xiushui2 | 28.80 /114.74 | | Ct1 | 7 |  | | Ss1 | | 1 |  | Cp2 | 8 |  | Sc21 Sc22 | 5 |  | Ec5Ec11 | 9 |
| 55 | Yichun | 27.58 /114.38 | | — | — |  | | — | | — |  | — | — |  | — | — |  | — | — |
| 56 | Yudou | 25.64 /115.33 | | Ct4 | 6 |  | | Ss2 Ss3 | | 12 |  | — | — |  | Sc1Sc14 | 5 |  | — | — |
| 57 | Yihuang1 | 27.20 /116.04 | | — | — |  | | — | | — |  | Cp2 | 9 |  | Sc1Sc14 | 6 |  | — | — |
| 58 | Yihuang2 | 27.27 /116.34 | | Ct20 | 6 |  | | Ss5 Ss3 | | 7 |  | — | — |  | Sc1Sc18 | 5 |  | Ec10Ec14 | 4 |
| 59 | Yihuang3 | 27.38 /116.08 | | — | — |  | | — | | — |  | — | — |  | Sc1 | 6 |  | — | — |
| 60 | Guanshan | 28.53 /114.73 | | Ct1 | 9 |  | | — | | — |  | Cp2 Cp7 | 8 |  | Sc1 | 6 |  | Ec1 | 10 |
| 61 | Yanshan1 | 28.07 /117.59 | | Ct2Ct21 Ct22 | 7 |  | | Ss8 | | 8 |  | — | — |  | Sc1 | 9 |  | — | — |
| 62 | Yanshan2 | 28.11 /117.70 | | — | — |  | | Ss1 Ss5 | | 10 |  | Cp2Cp11 | 10 |  | — | — |  | Ec5 Ec10 | 3 |
| 63 | Yongfeng | 26.87 /115.77 | | Ct1 Ct19 | 8 |  | | — | | — |  | — | — |  | Sc1 | 4 |  | Ec10 | 10 |
| 64 | Yiyang | 28.21 /117.42 | | Ct18 Ct23Ct24Ct25 | 7 |  | | Ss8 | | 8 |  | — | — |  | Sc1 | 8 |  | — | — |
| 65 | Yongxiu1 | 29.31 /115.66 | | — | — |  | | — | | — |  | — | — |  | Sc21 | 6 |  | — | — |
| 66 | Yongxiu2 | 29.32 /115.49 | | — | — |  | | — | | — |  | — | — |  | Sc1Sc23 | 12 |  | — | — |
| 67 | Zixi1 | 27.67 /117.17 | | Ct12 | 8 |  | | Ss5 | | 8 |  | — | — |  | Sc1 | 6 |  | — | — |
| 68 | Zixi2 | 27.62 /116.19 | | Ct2Ct3 | 10 |  | | Ss5 | | 10 |  | Cp6 | 1 |  | Sc1 | 5 |  | — | — |
| Total |  |  |  |  | 39(262) |  | |  | | 44(364) |  |  | 26(194) |  |  | 58(417) |  |  | 20(127) |
